# Personal, Social, and Environmental Correlates of Walking to School Behaviors: Case Study in Austin, Texas

**DOI:** 10.1100/tsw.2008.63

**Published:** 2008-09-21

**Authors:** Xuemei Zhu, B. Arch, Chanam Lee

**Affiliations:** Department of Architecture and Department of Landscape Architecture and Urban Planning, College of Architecture, Texas A&M University, College Station, TX 77843-3137, USA

**Keywords:** walking, school, environment, health, disparity

## Abstract

Walking is an affordable and environmentally clean mode of transportation that can bring additional benefits as healthy physical activity. This cross-sectional study examines the prevalence and correlates of walking to or from school in eight elementary schools in Austin, Texas, which have high percentages of low-income, Hispanic students. A survey of 1,281 parents was conducted, including questions about personal, social, and environmental factors that may influence their decisions on the children's school transportation. Binary logistic regressions were used to estimate the odds of choosing walking as the children's typical school travel mode. The results showed that walking was a typical mode for 28 and 34% of trips to and from school, respectively, and mostly accompanied by an adult. Parents' education level, family's car ownership, children's and parents' personal barriers, and having the school bus service reduced the likelihood of walking, while positive peer influences encouraged walking. Among the physical environmental factors, living close to school was the strongest positive predictor; safety concerns and the presence of highway or freeway en route were negative correlates. We concluded that the location of school is a key, as it determines the travel distance and the presence of highway or freeway en route. In addition to environmental improvements, educational and other assistance programs are needed for both parents and children to overcome their personal barriers and safety concerns. Health and disparity issues require further attention, as many underprivileged children have no other means of school transportation but walking in unsafe and poor environments.

### INTRODUCTION

Walking is an affordable and environmentally clean mode of transportation that can bring physical, psychological, and social health benefits for schoolchildren[1]. The increasing rate of childhood obesity[2] calls for more attention to such routine, daily physical activities[3,4,5]. In reality, however, the percentage of children who walk or bike to school has decreased dramatically from 41% in 1969 to 13% in 2001[6].

Various factors may have contributed to the decline in walking, including personal, social, and physical environmental factors[6,7,8,9,10,11,12,13,14,15,16,17,18]. Based on the socioecological theory[19] and previous literature, a conceptual framework is proposed for this study (see Fig. 1). The outcome variable is whether walking is a typical commuting mode to or from school. Personal factors include sociodemographic characteristics (such as the child's age, gender, and ethnicity; parents' education levels and marital status; and the household's car ownership) and the child's and parents' walking behaviors, attitudes, and barriers. Social factors include influences from the school (such as the availability of school bus services) and other children and parents (including their school travel modes and walking behaviors). Physical environmental factors include walkability and safety en route to school. Walkability can be captured through the distance to school, sidewalk quality, overall walking environment (such as maintenance, tree shade, and visual quality), physical barriers (such as highways and busy streets), and land use conditions.

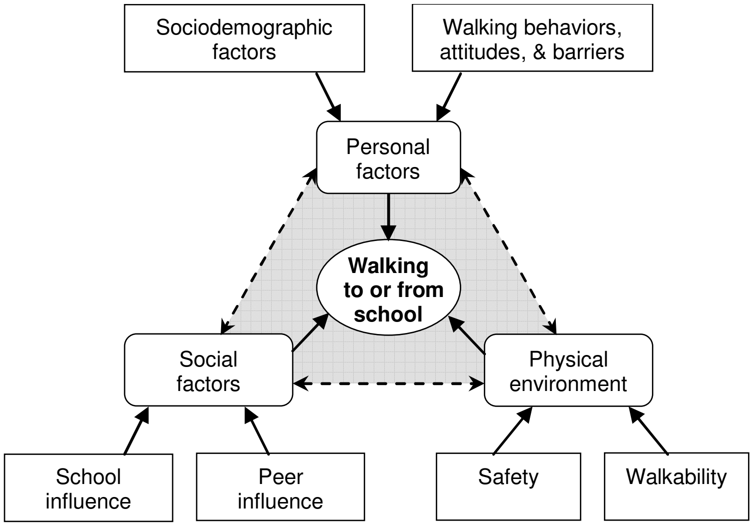

**FIGURE 1.** Conceptual framework on children's walking to or from school behaviors.

This study focuses on the schools with high percentages of low-income families and Hispanic students. The literature has shown that low-income minority children might walk more often in their school travels[9,20,21], but often do so in poor and unsafe environments[22,23]. In such a case, the potential threats to personal safety and respiratory health might outweigh the benefits of walking as physical activity. It also implies the possibility that these populations have their specific concerns in school travels. However, such disparity-related issues have been understudied in previous literature. This cross-sectional study focuses on low-income Hispanic children who are at high risk of developing obesity[2] and examines the prevalence and correlates of walking as a typical school travel mode. It also discusses the health and equity implications of findings from the study.

### METHODS

#### Study Areas and Previous Findings

The schools for this study were selected from the Austin Independent School District (AISD) in the city of Austin, Texas, which is the state's capital with an estimated population of 678,457 persons in 2005[24]. Like many other Texas cities, Austin features a high percentage of Hispanics or Latinos (32.9%

in 2005). The AISD covers the majority of the city's central area and had 81,003 students in the 2005–2006 school year, among whom 55.4% were Hispanics and 60.3% were economically disadvantaged (eligible for free or reduced-price lunch based on household income and size)[25]. Our previous study in AISD examined the entire 73 elementary schools' attendance areas to explore disparities in the environmental support for walking[22]. Geographic information systems (GIS) were used to measure the neighborhood-level walkability and safety; field audits were conducted to assess the street-level walkability. Analyses of variance and regression results showed the existence of economic and ethnic disparities. Schools with lower economic status had much lower street-level walkability (captured as visual quality, physical amenities, and perceived safety, etc.), but had two favorable conditions — shorter distances to school and lower traffic volumes — at the neighborhood level. Schools with higher percentages of Hispanic students were exposed to greater dangers from traffic and crime, but had better neighborhood-level walkability with shorter distances to school, more completed sidewalk networks, and greater land-use mix.

Building onto these findings on the aggregated environmental correlates, this study examines individual survey data from the individual students' parents to better understand their considerations regarding the children's school transportation modes. Further, noting the gaps in the existing literature, this study selected eight elementary schools with low economic status (over 90% of students from economically disadvantaged households) and high percentages of Hispanic students (65.8–96.5%). However, it was ensured that the physical environmental features of the selected schools' administrative areas had sufficient variations, including crash and crime rates and students' distances from school (Table 1). Fig. 2 shows the Hispanic student percentage of each elementary school in AISD and the locations of the selected schools.

**TABLE 1**
**Background Information of Selected Schools Compared to the Mean of all Elementary Schools in the AISD**

| Group of Schools | Name of School | Total Enrollment | Hispanic Students (%) | Students Receiving Free or Reduced-Price Lunch (%) | Yearly Crash Rate per Street Mile | Yearly Crime Rate per 1,000 Persons | Students Living within 1 Mile of School (%) |
|---|---|---|---|---|---|---|---|
| Group 1 | Zavala | 460 | 88.9 | 93.5 | 6.8 | 507 | 87.7 |
| | Sanchez | 667 | 90.4 | 90.9 | 9.4 | 501 | 36.1 |
| | Metz | 661 | 96.5 | 91.5 | 12.3 | 315 | 36.1 |
| Group 2 | Harris | 688 | 78.6 | 97.8 | 5.3 | 218 | 91.0 |
| | Blanton | 617 | 77.8 | 93.0 | 3.9 | 258 | 69.7 |
| | Andrews | 600 | 65.8 | 93.2 | 4.4 | 161 | 36.3 |
| Group 3 | Wooten | 596 | 86.1 | 95.6 | 6.2 | 228 | 63.3 |
| | McBee | 820 | 75.7 | 94.4 | 10.4 | 146 | 33.8 |
| Mean of all elementary schools | | 605 | 59.5 | 68.6 | 4.7 | 239 | 49.9 |

Data sources: Texas Education Agency, Austin Independent School District, Austin Police Department.

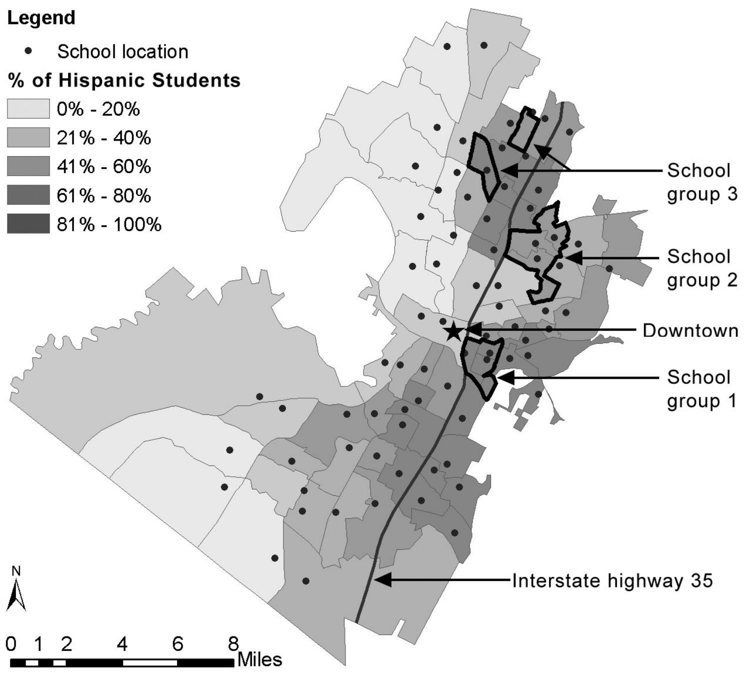

**FIGURE 2.** Percentage of Hispanic students within elementary schools in the AISD and locations of sampled schools.

According to the geographic locations, these eight schools are classified into three groups. Group 1 includes Zavala, Sanchez, and Metz, which are centrally located within the city, east of the downtown area across Interstate Highway (IH) 35. They feature small attendance areas, grid-like street networks, and small street blocks and land parcels. Harris, Blanton, and Andrews belong to Group 2, having larger attendance areas, cul-de-sac street networks, and larger street blocks and land parcels. Group 3 consists of Wooten and McBee, which are located farther north and west of IH 35 and feature a combination of cul-de-sac and superblock, grid-like street networks. Within each group, schools share relatively similar characteristics in terms of sociodemographic and physical environmental characteristics.

### Survey

This study focuses on the individual parent's perception of the physical environment, as well as the personal and social factors. The survey was administered in collaboration with the city's Child Safety Program and the AISD, as part of the city's efforts to create a Safe Routes to School (SRTS) Plan. The survey instrument was developed based on the conceptual framework, literature review, and several previously validated instruments, including the questionnaire from the University of California at Irvine's SRTS study[18], the Parent-Adolescent Survey[26], and the PedsQL Family Information Form[27]. After cognitive interviews and revisions, the final three-page questionnaire was developed to capture the child's typical school travel mode and the potential correlates including personal, social, and physical environmental factors. Most perception variables were measured using a five-point Likert scale by asking how much the respondent agreed or disagreed with each statement. Other variables were captured through binary measures (such as the presence of certain land uses, 0 = No, 1 = Yes) or categorical values (such as ethnicity, parents' education levels, and the household's car ownership).

In late April 2007, with the help from the teachers in the eight selected schools, a total of 4,759 surveys (including both Spanish and English versions) were sent out to the parents of all students as an item inserted into the weekly folio that students take home to their parents. A total of 1,281 valid responses were returned, yielding an average response rate of 26.9%, ranging from 17.9 to 42.8% across schools. Respondents' ethnicity and children's grade levels were tested to check the potential nonresponse bias. Ethnic composition of the respondents seemed comparable with the entire population, except for the Group 2 schools, where Hispanics were slightly over-represented and African Americans were somewhat under-represented. The 5th-grade students were slightly under-represented in the sample, but all other grades were well represented.

### Data Analysis

The mode share and travel time was examined for the pooled data from all eight schools and for each school separately. Binary logistic regressions were estimated between each individual independent variable and the outcome variable, one pair at a time. Following these regressions, an exploratory factor analysis was conducted for data reduction purposes and also for capturing the perception and attitude variables more effectively. Further, bivariate correlations among independent variables were analyzed, using Pearson correlations, analyses of variance, or binary logistic regressions, depending on the variable type. This step helped to examine multicollinearity issues for the following multiple regressions. Disparity issues underlying the environmental support for walking were also explored using bivariate analyses. In addition, mean comparisons were conducted across schools for comparing differences in the individual physical environmental variables.

For the final multivariate analyses, binary logistic regressions were used to predict the outcome variable (walking as a typical school-commuting mode) with personal, social, and physical environmental variables. A pooled regression model was estimated first using the total sample from all eight schools, with each school entered as an indicator (dummy) variable. Then, individual regression models were performed for each school separately to examine the differences or similarities in the predictors of walking among the schools.

## RESULTS

### Preliminary Analysis

From the bivariate analysis with sociodemographic variables, only the parents' highest education level showed a significant negative correlation with walking to or from school (odds ratio [OR] = 0.882). However, all variables were retained in the following analysis due to their theoretical importance. For the personal attitude variables, social factors, and physical environmental variables, 11 of 52 variables showed insignificant bivariate correlations ($p > 0.1$) and were excluded from further analyses. For the remaining ones, missing values for the continuous variables were tested for their randomness and then imputed using the mean of the corresponding school.

From the factor analysis, seven latent factors were extracted from the remaining continuous observed variables. They included (1) child's personal barriers, (2) parent's personal barriers, (3) both child's and parents' walking behaviors and attitudes, (4) positive peer influences, (5) safety concerns, (6) sidewalk quality, and (7) quality of overall walking environments. Consequently, the total number of independent variables was reduced from 58 to 21 (Table 2).

### Mode Share and Travel Time

From the pooled data of all eight schools, walking was a typical commuting mode for 28 and 34% of trips to and from school, respectively (Table 3). This is significantly higher than the national survey of 5- to 18-

**TABLE 2**
**Predictors of Walking To or From School, Unadjusted[a]**

| Predictors | Individual Observed Variables or Coding Scheme | Association[b] |
|---|---|---|
| **Personal Factors** | | |
| Child's grade level | | × |
| Child's gender | (0 = female, 1 = male) | × |
| Child's ethnicity | (0 = non-Hispanic, 1 = Hispanic) | × |
| Parents' highest education level | (1 = 6th grade or less; …; 7 = graduate or professional degree) | –** |
| Parents' marital status | (Single-parent or not: 0 = no, 1 = yes) | × |
| Household's car ownership | | × |
| Child's personal barriers *(factor)* | 1. "My child has too much to carry." | –** |
| | 2. "My child gets too hot and sweaty." | (–) |
| Parents' personal barriers *(factor)* | 1. "I have no time to walk with my child to/from school." | –** |
| | 2. "It is easier for me to drive my child to/from school." | –** |
| Child's and parents' positive walking behaviors and attitudes *(factor)* | 1. "My child thinks walking to school is 'cool'." | +** |
| | 2. "My child walks quite often in his/her daily routine." | +** |
| | 3. "Walking is a good way to interact with other people." | +** |
| | 4. "I walk quite often in my daily routine." | +** |
| | 5. "I enjoy walking with my child to/from school." | +** |
| | 6. "My family and friends like the idea of walking to school." | +** |
| **Social Factors: School and Peer Influence** | | |
| Having school bus service | (0 = no, 1 = yes) | –** |
| Positive peer influences *(factor)* | 1. "Other kids walk to/from school." | +** |
| | 2. "Other kids walk quite often in their daily routines." | +** |
| | 3. "Other parents walk quite often in their daily routines." | +** |
| **Built Environment: Perceived Safety and Walkability** | | |
| Safety concerns *(factor)* | 1. "My child may get lost." | –** |
| | 2. "My child may be taken or hurt by a stranger." | –** |
| | 3. "My child may get bullied, teased, or harassed." | –** |
| | 4. "My child may be attacked by stray dogs." | –** |
| | 5. "My child may be hit by a car." | –** |
| | 6. "Exhaust fumes will harm my child's health." | –** |
| Distance to school | "Is the distance close enough…?" (0 = no, 1 = yes) | +** |
| Physical barriers | "Does your child have to cross the following on the route …?" | |
| Highway or freeway | (0 = no, 1 = yes) | –** |
| Road with busy traffic | (0 = no, 1 = yes) | –** |
| Sidewalk quality *(factor)* | 1. "Sidewalks are well maintained and clean." | +* |
| | 2. "Sidewalks are wide enough." | +** |
| | 3. "Sidewalks are separated from traffic by grass/trees." | +* |

*(Table 2 continues)*

**TABLE 2 (continued)**

| | | |
|---|---|---|
| Walking environment *(factor)* | 1. "It is convenient to walk to school." | +** |
| | 2. "It is well maintained and clean." | +** |
| | 3. "It is well shaded by trees." | (+) |
| | 4. "It is quiet." | +** |
| | 5. "Streets are well lit." | +** |
| Land uses and public transit en route to school | | |
| Convenience store | (0 = no, 1 = yes) | –** |
| Office building | (0 = no, 1 = yes) | –** |
| Vacant site | (0 = no, 1 = yes) | –** |
| Bus stop | (0 = no, 1 = yes) | –** |

[a] This table presents results from simple bivariate analyses between individual independent variables and the outcome variable, one pair at a time, without controlling for other variables. All continuous variables were measured on a five-point Likert scale ranging from "1 = strongly disagree" to "5 = strongly agree". For sidewalk-related questions, "0" was used when there was no sidewalk.

[b] ×, Insignificant association; –, negative association; +, positive association; results in parentheses are marginally significant at the 0.1 level; *$p < 0.05$; **$p < 0.01$.

**TABLE 3**
**Travel Mode and Travel Time of the Pooled Sample from Eight Schools**

| Travel Mode | Trip from Home to School (n = 1271) | | | | Trip from School to Home (n = 1201) | | | |
|---|---|---|---|---|---|---|---|---|
| | Mode Share | Travel Time by Mode (Minutes) | | | Mode Share | Travel Time by Mode (Minutes) | | |
| | | <15 | 15–30 | >30 | | <15 | 15–30 | >30 |
| Walk alone | 2.0% | 73.9% | 26.1% | 0% | 2.6% | 66.7% | 33.3% | 0% |
| Walk with friends | 4.6% | 69.6% | 25.0% | 5.4% | 7.8% | 76.9% | 17.6% | 5.5% |
| Walk with a parent/adult | 21.4% | 71.4% | 23.7% | 4.9% | 23.6% | 68.5% | 27.8% | 3.7% |
| Bike | 0.6% | 71.4% | 28.6% | 0% | 0.6% | 75.0% | 25.0% | 0% |
| School bus | 25.6% | 63.0% | 32.9% | 4.1% | 26.7% | 63.1% | 33.6% | 3.3% |
| Public bus | 2.9% | 35.5% | 35.5% | 29.0% | 3.5% | 40.0% | 30.0% | 30.0% |
| Private car | 42.8% | 88.1% | 10.8% | 1.1% | 35.2% | 87.2% | 11.1% | 1.7% |

year-old children that showed that only 17% walked to or from school at least once per week in a usual week[7]. One possible reason is the low socioeconomic status of the selected populations (and correspondingly low car ownership) and the urban context of this study. What is also important is that most walking students were accompanied by a parent or guardian while walking. Biking was rarely used among these elementary school children. School bus ridership accounted for about 26% of the mode share. It should be mentioned that the AISD provides bus services only for the students living farther than 2 miles from school and those who live within 2 miles, but have to face hazardous conditions, such as a highway or freeway on the trips to school. In addition, the school-to-home trip had a slightly higher walking rate than the home-to-school trip, which is consistent with previous studies[12].

Average travel times for all modes were similar except for public transit, which was longer than the others (Table 3). Private cars took slightly less time than the other modes on average. The majority (63.0–88.1%) of these modes (except public bus) took less than 15 min to get to or from school. From each school's individual data (Table 4), some variations in the mode share were observed. For example, the rates of walking ranged from 20.5 to 47.6%. From the adjusted mode share that excludes the school bus riders, walking ranged from 30.9 to 50.4%.

**TABLE 4**
**Mode Share for Each School (Trips To and From School Combined)**

| Travel Mode | School Group 1 | | | School Group 2 | | | School Group 3 | |
|---|---|---|---|---|---|---|---|---|
| | Zavala | Sanchez | Metz | Harris | Blanton | Andrews | Wooten | McBee |
| Walk | 47.6% | 22.3% | 26.0% | 44.8% | 20.5% | 24.5% | 38.4% | 30.7% |
| Bike | 0.9% | 0.7% | 0.3% | 0.0% | 0.8% | 0.0% | 1.5% | 0.7% |
| School bus | 5.6% | 39.4% | 37.6% | 1.7% | 40.2% | 46.8% | 18.7% | 0.7% |
| Public bus | 2.3% | 1.0% | 3.5% | 5.4% | 0.8% | 3.9% | 1.0% | 7.9% |
| Private car | 43.5% | 36.8% | 32.5% | 48.1% | 37.7% | 25.0% | 40.4% | 60.1% |
| *Walk adjusted*[a] | *50.4%* | *36.8%* | *41.7%* | *45.6%* | *34.3%* | *46.1%* | *47.2%* | *30.9%* |

[a] The adjusted walking rate was calculated after excluding school bus riders.

### Correlations Among Independent Variables

Bivariate correlations among independent variables showed that multicollinearity would not be a problem for the following multiple regressions. Meanwhile, they provided interesting insights into the disparity issues. The parents' highest education level was positively associated with car ownership and safety concerns, and negatively associated with the quality of overall walking environments. Because education could be considered as a proxy of the family's economic status, this finding appeared contradictory to our previous finding that schools in higher-income neighborhoods had better street-level walkability and safety in their administrative areas. The reasons might be the differences in the units of analysis (the aggregated attendance area vs. the disaggregated, individual home-to-school route) and in the measurement type (objective vs. subjective measures). One may also suspect that parents with higher education levels have higher expectations for their environment and greater awareness about the safety problems, consequently increasing their safety concerns and reliance on cars for their children's school travel. This was further supported by the positive association between the car ownership and parents' perceived barriers to letting their children walk.

### Mean Comparisons of Physical Environmental Variables Across Schools

Analyses of variance and binary logistic regressions were used to compare means of physical environmental variables across schools, and to explore disparities in the environmental support for walking. Safety concerns showed no significant differences, which was somewhat surprising considering the clear differences in these schools' objective measures of safety based on the crash and crime rates (Table 1). One possible explanation is that parents' perceptions tend to overestimate the safety threats to children no matter what the factual data show. In addition, McBee, as the only school that is remotely located in a suburban neighborhood, showed poorer sidewalk quality and overall walking environments than most other schools. More parents from McBee reported busy roads to be barriers than those from other schools.

### Predicting Walking Using Personal, Social, and Environmental Variables

#### *Regression Model for the Pooled Data of Eight Schools*

A binary logistic regression was fitted using the pooled data from all eight schools to identify the personal, social, and physical environmental correlates of walking to or from school (Table 5). A set of dummy variables for the school membership was added to this model to help account for the clustering effect of the students attending the same school. Overall, the model explained about 46% of the variance in walking.

Among the personal factors, the parents' highest education level was inversely correlated with walking after controlling for the other factors. The education variable was measured using a scale from "1" (6th grade or less) to "7" (graduate or professional degree). With one-unit increase in the education level, the child was about 19% less likely to walk to or from school. A similar inverse relationship was found for car ownership (OR = 0.789). Two factor variables were also found to be significant. Parental barrier factor was negatively associated with walking (OR = 0.566), while the factor capturing children's and parents' positive walking behaviors and attitudes was a positive correlate (OR = 1.461).

Among the social factors, students attending Blanton elementary school were less likely to walk than were students from other schools (OR = 0.324). In addition, having school bus services lowered the odds of walking by 67%. The factor of positive peer influences ("other kids walk to/from school" and "other kids/parents walk often in their daily routines" loaded to the factor) increased the odds of walking by 19%.

For the physical environmental variables, distance to school was the strongest predictor, where the child was about four times more likely to walk if the parent perceived the distance to be close enough for their child to walk. This is consistent with previous findings where 61.5% of the parents reported distance to be a barrier to their children's walking-to-school behaviors[7], which was increased from 55% in a similar study conducted in 1999[28]. In addition, one-unit increase in the safety concern factor (ranged from −2.6 to 1.9) reduced the odds of walking by 22%. Further, the presence of highway or freeway barrier decreased the likelihood of walking by 52%. The findings about land uses, however, were somewhat surprising. Previous studies showed that mixed land uses had inconsistent relationships with walking to school. In this study, the presence of convenience stores and office buildings was associated with decreased likelihood of walking after controlling for other variables. Moreover, the sidewalk quality factor and overall walking environment factor did not show significant associations with walking. This could be potentially due to the insufficient variability in these two measures.

#### *Regression Models for Individual Schools*

Eight separate models were estimated for individual schools. Overall, fewer variables were found significant in the individual models, probably because of the smaller sample size and reduced variations in some of the independent variables. According to the pseudo-$R^2$ values, all individual models (except Harris) achieved better model fit (ranging from 0.462 to 0.682) compared to the pooled model (0.459).

Table 6 lists significant ORs from eight individual models, showing some consistent patterns as well as a few clear differences from the pooled model. The distance to school, again, was the most significant predictor of walking in six of eight schools, with the ORs ranging from 7.467 for Zavala to as high as 11.735 for Sanchez. The parental barrier factor was the second most important correlate showing significance in five schools, and the ORs ranged from 0.183 to 0.593. Other important variables included school bus service, safety concerns, car ownership, walking behaviors and attitudes, and the presence of highway or freeway en route to school.

**TABLE 5**
**Binary Logistic Regression for the Pooled Sample from Eight Schools (n = 1180)**

| Independent Variables | Coefficient (β) | Standard Error | Odds Ratio |
|---|---|---|---|
| **Personal Factors** | | | |
| Child’s grade level | _[a] | _ | _ |
| Child’s gender (0 = female, 1 = male) | _ | _ | _ |
| Hispanic ethnicity (0 = no, 1 = yes) | _ | _ | _ |
| Parents’ highest education level (range: 1 – 7) | –0.207 | 0.058 | 0.813** |
| Single-parent status (0 = no, 1 = yes) | (–0.295)[b] | (0.176) | (0.745) |
| Household’s car ownership | –0.238 | 0.087 | 0.789** |
| Child’s personal barriers *(factor)* | _ | _ | _ |
| Parents’ personal barriers *(factor)* | –0.569 | 0.085 | 0.566** |
| Child’s and parents’ positive walking behavior and attitude *(factor)* | 0.379 | 0.079 | 1.461** |
| **Social Factors: School and Peer Influence** | | | |
| School membership: | | | |
| 1. Andrews | _ | _ | _ |
| 2. Blanton | –1.127 | 0.382 | 0.324** |
| 3. Harris | _ | _ | _ |
| 4. McBee | _ | _ | _ |
| 5. Metz | _ | _ | _ |
| 6. Sanchez | (–0.686) | (0.358) | (0.504) |
| 7. Wooten | _ | _ | _ |
| Having school bus service (0 = no, 1 = yes) | –1.100 | 0.199 | 0.333** |
| Positive peer influence *(factor)* | 0.171 | 0.087 | 1.187* |
| **Built Environment: Perceived Safety and Walkability** | | | |
| Safety concerns *(factor)* | –0.253 | 0.079 | 0.776** |
| Distance close enough (0 = no, 1 = yes) | 1.593 | 0.174 | 4.918** |
| Physical barrier: | | | |
| Highway or freeway (0 = no, 1 = yes) | –0.727 | 0.236 | 0.483** |
| Busy road (0 = no, 1 = yes) | _ | _ | _ |
| Sidewalk quality *(factor)* | _ | _ | _ |
| Quality of overall walking environment *(factor)* | _ | _ | _ |
| Presence of land uses or public transit en route: | | | |
| Convenience store (0 = no, 1 = yes) | –0.531 | 0.188 | 0.588** |
| Office building (0 = no, 1 = yes) | –0.654 | 0.267 | 0.520* |
| Vacant lot (0 = no, 1 = yes) | _ | _ | _ |
| Bus stop (0 = no, 1 = yes) | _ | _ | _ |

[a] _, Insignificant.

[b] Results in parentheses are marginally significant at the 0.1 level. *$p < 0.05$; **$p < 0.01$.

**TABLE 6**
**Significant Odds Ratios from Individual Binary Logistic Regression Models for Each School**

| Independent Variables | School Group 1 | | | School Group 2 | | | School Group 3 | |
|---|---|---|---|---|---|---|---|---|
| | Zavala (n = 106) | Sanchez (n = 150) | Metz (n = 153) | Harris (n = 117) | Blanton (n = 114) | Andrews (n = 215) | Wooten (n = 193) | McBee (n = 137) |
| **Personal Factors** | | | | | | | | |
| Child's grade level | 1.477* | _[a] | _ | (1.283)[b] | _ | _ | _ | _ |
| Child's gender (1 = male) | _ | _ | _ | _ | _ | _ | _ | (0.388) |
| Hispanic ethnicity (1 = yes) | | | | _ | _ | (0.258) | | _ |
| Parents' education level (range: 1–7) | _ | (0.610) | _ | _ | _ | 0.637* | _ | (0.716) |
| Single-parent status (1 = yes) | _ | 0.142** | (0.307) | _ | _ | _ | _ | _ |
| Household's car ownership | _ | 0.369* | (0.481) | _ | _ | _ | 0.463** | _ |
| Child's barriers *(factor)* | _ | _ | _ | _ | _ | _ | _ | _ |
| Parents' barriers *(factor)* | 0.183** | _ | 0.453* | 0.593* | _ | 0.436** | (0.687) | 0.354** |
| Child's and parents' positive walking behavior and attitude *(factor)* | _ | _ | _ | 2.160** | _ | _ | _ | 2.300** |
| **Social Factors: School and Peer Influence** | | | | | | | | |
| Having school bus service (1 = yes) | | (0.247) | 0.169** | | _ | _ | 0.309* | |
| Positive peer influence *(factor)* | _ | _ | _ | _ | _ | _ | _ | _ |
| **Built Environment: Perceived Safety and Walkability** | | | | | | | | |
| Safety concerns *(factor)* | 0.352** | _ | 0.265** | _ | _ | (1.735) | _ | 0.419** |
| Distance close enough (1 = yes) | 7.467* | 11.735** | 9.177** | _ | 10.384** | 11.680** | 9.441** | _ |
| Physical barrier: | | | | | | | | |
| Highway or freeway | | _ | | | | 0.108** | _ | _ |
| Busy road | _ | _ | (3.871) | _ | 0.203* | _ | _ | _ |
| Sidewalk quality *(factor)* | _ | _ | _ | 0.477* | _ | (1.794) | _ | _ |
| Quality of overall walking environment *(factor)* | _ | _ | _ | _ | _ | _ | _ | _ |
| Presence of land uses and public transit en route (1 = yes): | | | | | | | | |
| Convenience store | (0.149) | _ | _ | 0.262* | _ | _ | _ | _ |
| Office building | 14.596* | _ | | _ | | | | _ |
| Vacant lot | _ | | (0.236) | | _ | | | _ |
| Bus stop | _ | _ | _ | _ | _ | _ | _ | _ |

[a] _, Insignificant

[b] Results in parentheses are marginally significant at the 0.1 level. *$p < 0.05$; **$p < 0.01$.

Four insignificant variables from the pooled model became significant in the individual models. Among the personal variables, age became significant in the Zavala model, with 1-year increase resulting in a 48% increase in the odds of walking. Single-parent status became significant in Sanchez, with children from single-parent families being much less likely to walk (OR = 0.142). From the physical environmental variables, the busy-road barrier showed a negative association with walking in Blanton (OR = 0.203). One unexpected result was found about the sidewalk quality factor, which showed a negative association with walking in Harris (OR = 0.477). One possible explanation could be that walkers' parents (many reported walking with their child) know more about their environments, such as the physical barriers and problems with sidewalk quality. Another speculation would be that people of lower socioeconomic status are more likely to walk for transportation[29,30] and also to live in areas that are unsafe and have poor infrastructure quality. Unfortunately, the income variable could not be used in the analysis to directly test this speculation, due to the large number of missing responses. Another interesting finding is that the presence of office buildings was positively correlated with walking in Zavala. Although consistency between these individual models and the pooled model are not expected, it is interesting to note that the same variable was a negative factor in the pooled model. We suspect that the unique environment of Zavala (located near the city's vibrant downtown) might explain this result. Unlike office buildings in suburban locations (usually developed with extensive surface parking and in large strip-center type arrangements located along major arterials), office buildings in downtown Austin are compact and designed with small or no surface parking, and located among diverse retail, commercial, or service uses.

### DISCUSSION

This study focused on the roles of the perceived built environment in promoting walking as a routine school travel mode through a case study in Austin, Texas. The impact of personal and social factors are also examined and revealed the complex and multilevel relationships among these factors. Several key findings are worth reiterating.

First, the travel distance is the strongest positive correlate of walking to or from school, and calls for new perspectives in future policies and practice related to school sitting. The most remotely located suburban school, McBee, showed significantly higher dependency on private cars (60.1%) compared to the other schools (34.3–50.4%). Further, multiple regression models confirmed "child living close enough to school" to be a significant factor after controlling for other variables, with high ORs ranging from 4.918 for the pooled sample to 11.735 for the Sanchez elementary.

Second, along with distance, parents consider safety to be another foremost important factor in choosing walking as their children's school transportation mode. The "perception" of safety shows no significant differences among schools, although the factual crime or crash data showed dramatic differences. This finding implies that parents may overexaggerate dangers from traffic and crime when it comes to children's school travel. In order to overcome such *psychological* safety concerns, educational and other assistance programs are needed, in addition to the physical improvements of environmental safety. Programs such as the "Walking School Bus" seem promising, as being accompanied by parents or other familiar adults on school trips may help to reduce safety concerns and increase social support. In addition, the "perception" of physical barriers, such as high-volume and high-speed roadways seems to be crucial for walking to or from school. Future school sitting should avoid locating schools next to highways or freeways or allocating a school's attendance area in a way that a highway or freeway runs directly through the middle of the area

Third, findings from this study revealed some health and equity issues related to school transportation. Parents' educational levels and family's car ownership were negatively correlated with walking after controlling for other factors, implying that children of lower economic status walk more often for school travel than their affluent peers. However, they must do so in poor-quality and unsafe environments. In such a case, walking may not be perceived as a healthy physical activity due to the potential threats to personal safety and respiratory health. It may also be considered as a socially stigmatized activity by populations of lower socioeconomic status who do not own a car[31]. Further, in the AISD, the availability of school bus service is decided by the student's home-to-school distance and the presence of a highway or freeway en route to school. This rule leads to a lower rate of school bus service for those low-income children who are living in inner-city neighborhoods and closer to school. The high crime and crash rates in these neighborhoods[22] are neglected during the decision-making process regarding school bus service, not to mention their limited car ownership. Such disparity issues are further exaggerated by the fact that these low-income, minority children have much higher risks for developing obesity and have less access to other physical activity facilities.

Finally, among nonenvironmental factors, parental characteristics are the key determinants of elementary children's school transportation. This study also showed that most students who walked to or from school did so with a parent or a guardian. Future promotion efforts can be more effective if they target parents as well as children. School transportation is not an independent choice, but is decided by multiple household and environmental characteristics. Future research should further explore the complex relationships among multilevel correlates of active school commuting, and thereby inform policy makers and practitioners to develop more effective intervention efforts.

### ACKNOWLEDGMENT

The authors would like to acknowledge the help for this study from Chris Moore and other personnel in the City of Austin, Texas and the Austin Independent School District. This manuscript is a modification from a section of the following book chapter: Lee, C. and Zhu, X. School transportation, health and equity: the role of built environments. In *Transportation Research Trends*. Inweldi, P.O., Ed. Nova Science, New York; in press.

## References

1. Jackson, R. and Tester, J. (2008) Environment shapes health, including children's mental health. *J. Am. Acad. Child Adolesc. Psychiatry* **47(2),** 129–131.
2. Ogden, C.L., Flegal, K.M., Carroll, M.D., and Johnson, C.L. (2002) Prevalence and trends in overweight among US children and adolescents, 1999-2000. *JAMA* **288(14),** 1728–1732.
3. Cooper, A.R., Andersen, L.B., Wedderkopp, N., Page, A.S., and Froberg, K. (2005) Physical activity levels of children who walk, cycle, or are driven to school. *Am. J. Prev. Med.* **29(3),** 179–184.
4. Mackett, R.L., Lucas, L., Paskins, J., and Turbin, J. (2005) The therapeutic value of children's everyday travel. *Transp. Res. Pt. A Policy Pract*. **39(2–3),** 205–219.
5. Tudor-Locke, C., Neff, L.J., Ainsworth, B.E., Addy, C.L., and Popkin, B.M. (2002) Omission of active commuting to school and the prevalence of children's health-related physical activity levels: the Russian Longitudinal Monitoring Study. *Child Care Health Dev.* **28(6),** 507–512.
6. McDonald, N.C. (2007) Active transportation to school - trends among US schoolchildren, 1969-2001. *Am. J. Prev. Med*. **32(6),** 509–516.
7. Martin, S. and Carlson, S. (2005) Barriers to children walking to or from school - United States, 2004 (reprinted from *MMWR*, **54,** 949–952, 2005). *JAMA* **294(17),** 2160–2162.
8. Environmental Protection Agency (2003) Travel and Environmental Implications of School Sitting. Washington, D.C.
9. Ewing, R., Schroeer, W., and Greene, W. (2004) School location and student travel - analysis of factors affecting mode choice. *Transport. Res. Rec. J. Transport. Res. Board* **1895,** 55–63.
10. Ewing, R. and Forinash, C.V., and Schroeer, W. (2005) Neighborhood schools and sidewalk connections: what are the impacts on travel mode choice and vehicle emissions? *Transport. Res. News.* **237,** 4–10.
11. McMillan, T., Day, K., Boarnet, M., Alfonzo, M., and Anderson, C. (2006) Johnny walks to school- does Jane? Sex differences in children's active travel to school. *Child. Youth Environ.* **16(1),** 75–89.
12. Schlossberg, M., Greene, J., Phillips, P.P., Johnson, B., and Parker, B. (2006) School trips - effects of urban form and distance on travel mode. *J. Am. Plan. Assoc*. **72(3),** 337–346.
13. Merom, D., Tudor-Locke, C., Bauman, A., and Rissel, C. (2006) Active commuting to school among NSW primary school children: implications for public health. *Health Place* **12(4),** 678–687.
14. Timperio, A., Ball, K., Salmon, J., Roberts, R., Giles-Corti, B., Simmons, D., et al. (2006) Personal, family, social, and environmental correlates of active commuting to school. *Am. J. Prev. Med.* **30(1),** 45–51.
15. Boarnet, M.G., Anderson, C.L., Day, K., McMillan, T., and Alfonzo, M. (2005) Evaluation of the California Safe Routes to School legislation - urban form changes and children's active transportation to school. *Am. J. Prev. Med.* **28(2),** 134–140.
16. Boarnet, M.G., Day, K., Anderson, C., McMillan, T., and Alfonzo, M. (2005) California's safe routes to school program - impacts on walking, bicycling, and pedestrian safety. *J. Am. Plan. Assoc.* **71(3),** 301–317.
17. Kerr, J., Rosenberg, D., Sallis, J.F., Saelens, B.E., Frank, L.D., and Conway, T.L. (2006) Active commuting to school: associations with environment and parental concerns. *Med. Sci. Sports Exerc*. **38(4),** 787–794.
18. McMillan, T.E. (2003) Walking and Urban Form: Modeling and Testing Parental Decisions about Children's Travel. University of California, Irvine.
19. McLeroy, K.R., Bibeau, D., Steckler, A., and Glanz, K. (1988) An ecological perspective on health promotion programs. *Health Educ. Behav*. **15(4),** 351–377.
20. Braza, M., Shoemaker, W., and Seeley, A. (2004) Neighborhood design and rates of walking and biking to elementary school in 34 California communities. *Am. J. Health Promot*. **19(2),** 128–136.
21. Evenson, K.R., Huston, S.L., McMillen, B.J., Bors, P., and Ward, D.S. (2003) Statewide prevalence and correlates of walking and bicycling to school. *Arch. Pediatr. Adolesc. Med*. **157(9),** 887–892.
22. Zhu, X. and Lee, C. (2008) Walkability and safety around elementary schools: economic and ethnic disparities. *Am. J. Prev. Med*. **34(4),** 282–290.
23. Green, R.S., Smorodinsky, S., Kim, J.J., McLaughlin, R., and Ostro, B. (2004) Proximity of California public schools to busy roads. *Environ. Health Perspect*. **112(1),** 61–66.
24. U.S. Census Bureau (2005) American Community Survey. [cited April, 2007]; Available from: http://www.census.gov/
25. Texas Education Agency. 2005–2006 Academic Excellence Indicator System. [cited April, 2007]; Available from: http://www.tea.state.tx.us/perfreport/aeis/2006/index.html
26. Kerr, J., Saelens, B., Rosenberg, D., Norman, G., Durant, N., Eggerman, J., et al. (2006) Active Where?: Multi-Region Formative Research to Understand Children’s Physical Activity Environments. 2006 Active Living Research Annual Conference, Coronado, CA.
27. Varni, J.W., Seid, M., and Kurtin, P.S. (2001) PedsQL (TM) 4.0: reliability and validity of the Pediatric Quality of Life Inventory (TM) version 4.0 generic core scales in healthy and patient populations. *Med. Care* **39(8),** 800–812.
28. Dellinger, A.M. and Staunton, C.E. (2002) Barriers to children walking and biking to school - United States, 1999 (Reprinted from *MMWR*, **51**, 701–704, 2002). *JAMA* **288(11),** 1343–1344.
29. Giles-Corti, B. and Donovan, R.J. (2002) Socioeconomic status differences in recreational physical activity levels and real and perceived access to a supportive physical environment. *Prev. Med*. **35(6),** 601–611.
30. Lee, C. and Moudon, A.V. (2006) Correlates of walking for transportation or recreation purposes. *J. Phys. Act. Health* **3(S1),** S77–S98.
31. Bostock, L. (2001) Pathways of disadvantage? Walking as a mode of transport among low-income mothers. *Health Soc. Care Community* **9(1),** 11–18.
